# Conservation of thermal physiology in tropical intertidal snails following an evolutionary transition to a cooler ecosystem: climate change implications

**DOI:** 10.1093/conphys/coad056

**Published:** 2023-08-01

**Authors:** David J Marshall, Nurshahida Mustapha, Cristián J Monaco

**Affiliations:** Environmental and Life Sciences, Faculty of Science, Universiti Brunei Darussalam, Jalan Tungku Link, Gadong, BE1410, Brunei Darussalam; Environmental and Life Sciences, Faculty of Science, Universiti Brunei Darussalam, Jalan Tungku Link, Gadong, BE1410, Brunei Darussalam; IFREMER, IRD, Institut Louis-Malardé, Univ Polynésie française, Tahiti, Polynésie française, EIO, F-98725 Taravao, France

**Keywords:** ecophysiology, gastropod, heat tolerance, thermal performance curve

## Abstract

Predictions for animal responses to climate warming usually assume that thermal physiology is adapted to present-day environments, and seldom consider the influence of evolutionary background. Little is known about the conservation of warm-adapted physiology following an evolutionary transition to a cooler environment. We used cardiac thermal performance curves (cTPCs) of six neritid gastropod species to study physiological thermal trait variation associated with a lineage transition from warmer rocky shores to cooler mangroves. We distinguished between functional thermal performance traits, related to energy homeostasis (*slope gradient, slope curvature, HR_max_*, maximum cardiac activity and *T_opt_*, the temperature that maximizes cardiac activity) and a trait that limits performance (*ULT*, the upper lethal temperature). Considering the theory of optimal thermal performance, we predicted that the functional traits should be under greater selective pressure to change directionally and in magnitude than the thermal limit, which is redundant in the cooler environment. We found little variation in all traits across species, habitats and ecosystems, despite a ~20°C reduction in maximum habitat temperature in the mangrove species over 50 million years. While *slope gradient* was significantly lowered in the mangrove species, the effect difference was negated by greater thermal plasticity in the rocky shore species. *ULT* showed the least variation and suggested thermal specialization in the warmest habitat studied. The observed muted variation of the functional traits among the species may be explained by their limited role in energy acquisition and rather their association with heat tolerance adaptation, which is redundant in the mangrove species. These findings have implications for the conservation of habitat of intertidal gastropods that transition to cooler environments. Furthermore, they highlight the significance of evolutionary history and physiological conservation when predicting species responses to climate change.

## Teaser

We report on limited variation in the thermal physiology of intertidal gastropods that underwent an evolutionary transition from warm rocky shores to cool mangroves. Thermal adaptation of these gastropods centres on heat tolerance, which becomes redundant in the mangrove species. This finding carries implications for conservation management and predicting species responses to climate warming.

## Introduction

Predicting how animals should respond to climate warming is a challenge for evolutionary and environmental biologists (Huey *et al.,* 2009, 2012; Terblanche *et al.,* 2011; Sunday *et al.,* 2012; Pacifici *et al.,* 2015; Huey and Kingsolver, 2019). Extensive consideration has been given to describing the thermal regimes of species, and determining behavioural and physiological responses to temperature change (Angilletta, 2009). While behavioural thermoregulation (the capability of ectothermic animals to modify their body temperature in response to acutely varying habitat temperature) and thermal acclimation (lifetime phenotypic changes) complicate understanding, these concepts are relatively well studied (Angilletta, 2009). Furthermore, using fundamental thermal niche theory, researchers have modelled spatial habitat temperatures and organismal body temperatures to understand likely future species distributions (Huey and Stevenson, 1979; Huey and Kingsolver, 1989; Huey *et al.,* 2012). Importantly, however, most approaches and frameworks assume that observed physiological performances and tolerances directly relate to thermal adaptation to contemporary environmental conditions (Huey and Kingsolver, 1989; Angilletta, 2009; Muñoz *et al.,* 2014; Sinclair *et al.,* 2016; Bodensteiner *et al.,* 2021). Very few studies have considered the influence of evolutionary histories of species and ancestral climates on thermal performance (see Bennett *et al.,* 2021). Trait canalization, whereby physiological phenotypes are insensitive to environmental change, arises when selection pressure to change is weak (Flatt, 2005). Canalization is seen in lineages that evolved in hot paleoclimates and have subsequently undergone ecological transitions to cooler habitats (see Flatt, 2005; Polgar *et al.,* 2015). We investigated change in the thermal physiology of closely-related, tropical intertidal gastropod species following a transition from a warmer rocky shore to a cooler mangrove environment.

**Figure 1:**
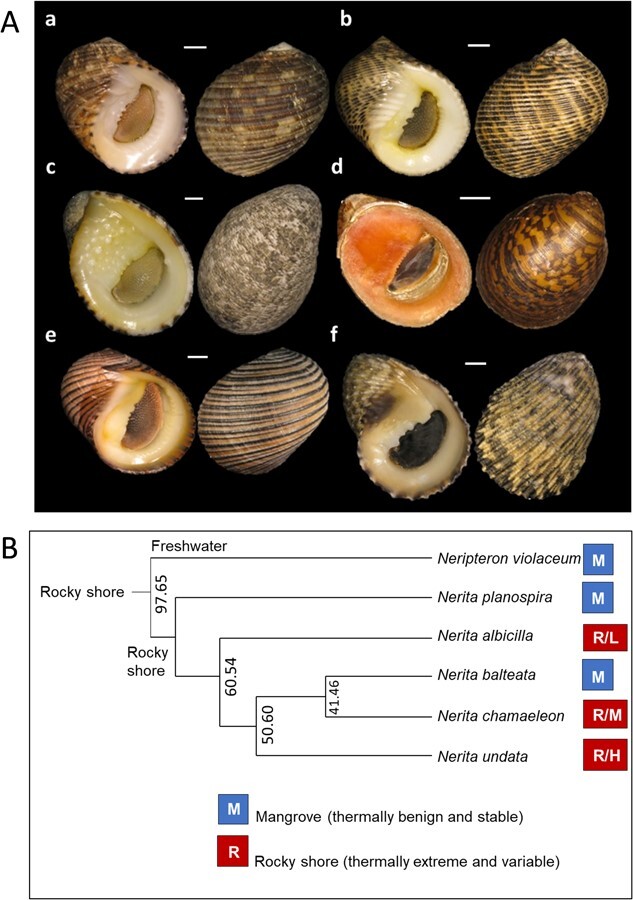
(A) The neritid species studied shown in apertural and abapertural view: a. *Nerita chamaeleon*, b. *N. undata*, c. *N. albicilla*, d. *Neripteron violaceum*, e. *N. balteata* and f. *N. planospira.* Scale bars represent 3 mm. (B) An abbreviated phylogeny of the species redrawn from Marshall *et al.* (2015), showing three independent transitions to mangroves (M). Rocky shore species occurred in the high shore (R/H), mid shore (R/M) and low shore (R/L). Numbers refer to divergence times (mya; Feng *et al.,* 2021). Details of other phylogenies are given in Frey and Vermeij (2008), Frey (2010), Wang *et al.* (2019) and Feng *et al.* (2020, 2021).

**Figure 2:**
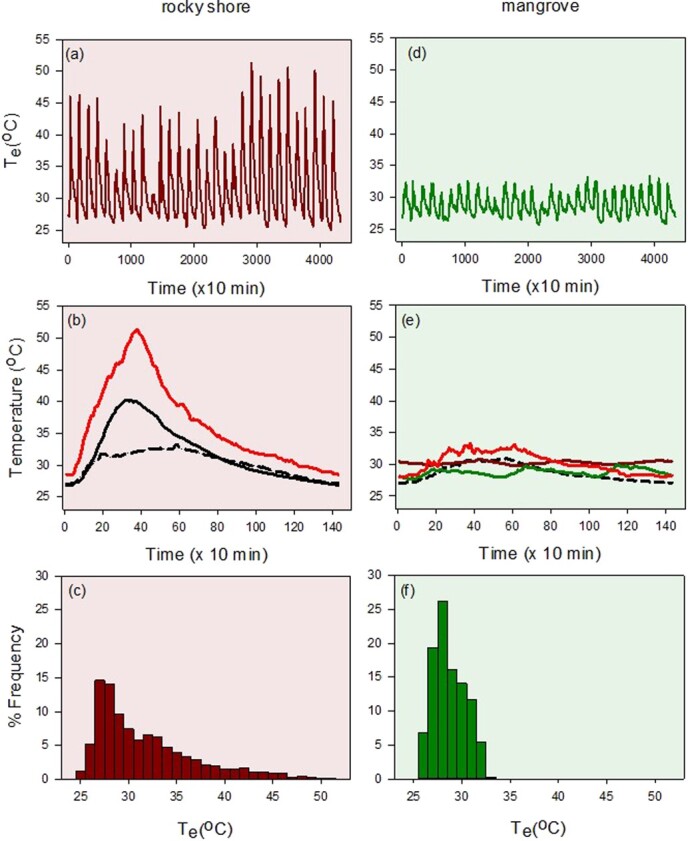
Environmental temperatures experienced by rocky shore and mangrove neritid snails (screened iButtons; Marshall *et al.,* 2015) in Brunei. (a, d) Temperature was recorded every 10 min for 30 days from upper rocky shore (sun-exposed, air) and mangrove trunk (shade, air). (b, e) Daily temperature variation averaged for 30 days for rocky shore, max (red), mean (solid) and shade (dashed) and for mangrove, trunk max (red), trunk mean (dashed), leaf mean (green) and mud mean (brown, tidally influenced). (c, f) Temperature frequency distributions based on (a, d); mean and maximum temperature for the rocky shore were 31.8 and 51.3°C (Δ = 19.5°C), and for mangroves were 27.7 and 33.2°C (Δ = 4.5°C). Water temperatures typically vary between 27 and 30°C.

Neritid snails are abundant and diverse in tropical intertidal zones (Marshall *et al.,* 2015). Phylogenetic analyses suggest that the contemporary species of this family have a common rocky shore ancestor, from which they have diversified and transitioned on multiple occasions over 50–100 million years to nutrient-rich mangrove ecosystems (Fig. 1; Frey and Vermeij, 2008; Frey, 2010; Marshall *et al.,* 2015; Wang *et al.,* 2019; Feng *et al.,* 2020, 2021). At least 16 species occur on the shorelines of Brunei, with *Nerita undata* Linnaeus, 1758, *Nerita chamaeleon* Linnaeus, 1758 and *Nerita albicilla* Linnaeus, 1758, being abundant in rocky shore habitats, and *Nerita planospira* Anton, 1838, *Nerita balteata* Reeve, 1855 and *Neripteron violaceum* Gmelin, 1791, being abundant in mangrove habitats (Fig. 1; Mustapha *et al.,* 2021; Mustapha and Marshall, 2021). The rocky-shore snails experience more extreme and variable temperatures, ranging between 27°C and 50°C, whereas local mangrove habitat temperatures (both sunned muddy surfaces or shaded trees) seldom rise above 35°C (Fig. 2; Marshall *et al.,* 2013, 2015). The mangrove species largely experience similar heat loading across habitats (muddy sediment, pneumatophores, shaded trees), whereas heat loading varies among the rocky shore species with vertical distribution in the following order: *N. albicilla* (low-shore)*, N. chamaeleon* (mid-shore) and *Nerita undata* (mid-high shore; Figs. 1 and 2; Marshall *et al.,* 2013, 2015).

**Figure 3:**
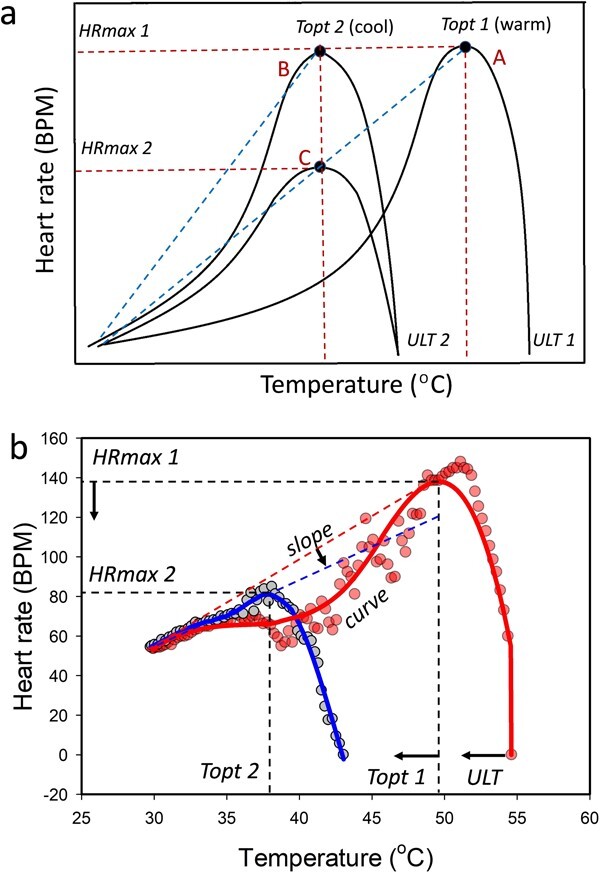
Predicted directional changes in cardiac traits with a transition from a warmer to a cooler marine intertidal environment. (a) Hypothetical cTPCs for a warm-adapted snail (A), a cool-adapted snail with *Topt* displaced towards cooler temperatures (B). (C) A cool-adapted snail with reduced *HRmax* as is observed in other intertidal gastropods (C). S*lope gradient* (shown here as a line drawn from *HRmin* to *Topt*; blue dashed line) may be similar or reduced and *slope curvature* is reduced in the cool-adapted snail. (b) The predictions are confirmed by cTPCs for a cool-adapted, low-rocky shore snail (*Trochus radiatus*; blue) and a hot-adapted, high-shore snail (*Echinolittorina vidua*; red). *ULT* is also reduced in the cooler-adapted snail. Data were plotted for two representative individuals from the same rocky shore and curves were fitted using negative exponential smoothing (Sigmaplot ver. 14) after correcting the data to a baseline of 50 BPM (see Monaco *et al.,* 2017).

Physiological and behavioural responses to temperature are complex, but can be described using thermal reaction norms, particularly the well-considered thermal performance curve (TPC; Sinclair *et al.,* 2016). Cardiac activity of marine gastropods indicates organismal physiological capabilities, informs about adaptive capacity, and conveys information about oxygen delivery and cellular energy demand (Marshall and McQuaid, 1992, 2011; Marshall *et al.,* 2011; Chen *et al.,* 2021; Liao *et al.,* 2021). Cardiac thermal performance curves (cTPCs) thus communicate functional aspects of physiology, such as the partitioning of energy to maintenance, growth, reproduction and thermoprotection (capacity adaptation), as well as communicating organismal tolerance (resistance adaptation) (Cossins and Bowler, 1987; Marshall *et al.,* 2011; Marshall and McQuaid, 2011; Liao *et al.,* 2021). Four functional attributes associate with the upslope of a cTPC. Maximum heart rate (*HRmax*) and the temperature at which heart rate is maximized (*Topt*) are primary traits, whereas *slope gradient* and *slope curvature* represent the interaction of these (Fig. 3a). The *slope gradient* increases when the thermal breadth contracts during displacement of a cTPC to a cooler thermal range (A versus B in Fig. 3a), but because *HRmax* is usually simultaneously reduced in cool-adapted marine gastropods (C in Fig. 3a), the *slope gradient* is often equal to or flatter than that of warm-adapted species (line C to A, Fig. 3a; Monaco *et al.,* 2017). *Slope curvature* captures heart rate variation at finer thermal increments than *slope gradient*, and is reduced by both contraction of the thermal breadth (A versus B in Fig. 3a) and depression of *HRmax* (A versus C in Fig. 3a). Many warm-adapted gastropods depress metabolic and heart rates during acute warming, further enhancing *slope curvature* (Fig. 3a; Marshall and McQuaid, 2011; Marshall *et al.,* 2011; Liao *et al.,* 2021). The temperature above the *Topt* that minimizes heart rate during acute warming (the flatline temperature) is synonymous with the upper lethal temperature (*ULT;*Marshall *et al.,* 2011; Ørsted *et al.,* 2022). Although more broadly framed functional traits, such as activity, growth and development are usually investigated (Chown, 2012; Sinclair *et al.,* 2016; Kellermann *et al.,* 2019), cTPC attributes imply physiological potential to support these higher-level functions. Also, the conventional term, critical thermal maximum (*CTmax*) inappropriately marks heat tolerance in intertidal gastropods, which cease locomotor activity at temperatures well below the *ULT* (Monaco *et al.,* 2017).

The above hypothesis for cTPC trait variation for warm and cool adapted animals is founded on the theory for optimality of performance and evolutionary fitness (see Angilletta, 2009). This hypothesis has also been shown to apply to rocky shore snails that inhabit warmer or cooler environments (Fig. 3b; Monaco *et al.,* 2017). In the present study, we used the six above-mentioned snail species *(N. undata, N. chamaeleon, N. albicilla, N. planospira, N. balteata* and *Neript. violaceum)* to test the prediction that directional trait change should accompany an eco-evolutionary transition from a warm rocky-shore to a cool mangrove environment. Specifically, we tested whether the values of all of five traits are lowered following this transition (Fig. 3; Monaco *et al.,* 2017). However, because the functional traits (*HRmax*, *slope gradient, curvature* and *Topt*) associate directly with organismal fitness (and impart evolutionary benefit) through energy homeostasis (the aquisition and utilization of energy), we further predicted that they should change more (greater selection strength) than the trait marking the performance limit (*ULT*), which is essentially redundant in the cooler mangrove environment. In other words, greater benefit should derive from a closer match between the functional physiology and the novel cooler thermal regime in the mangroves (capacity adaptation) than from losing an ancestral adaptation for heat tolerance (resistance adaptation).

The data presented are primarily intended to inform about pattern differences among the habitat-separated snails, rather than about evolutionary mechanisms, such as environmental canalization or evolutionary stasis. Inference on the latter would require information about both genetic and environmental drivers of the phenotypic variation (Burt, 2001; Flatt, 2005; Ellegren, 2010). We use simple terminology, such as trait conservation, where limited or no phenotypic variation is evident (Araújo *et al.,* 2013), and directional selection where the variation is appropriate to the direction of the environmental change (Burt, 2001; Flatt, 2005; Ellegren, 2010; Munoz *et al*. 2013). We also refer interchangeably to *attribute* and *trait*, with reference to a phenotypic feature that interacts with the environment. Such an evolutionary context for physiological trait variation should benefit climate warming frameworks, which usually assume a close match between thermal adaptation and contemporary climatic conditions (Angilletta, 2009).

## Materials and Methods

### Snail phylogeny and habitat temperatures

Multiple habitat transitions have occurred during the evolution of neritid snails, from rocky shores to mangroves, from lower to upper rocky-intertidal zones, and from hard to soft substrata (mud or sand) in mangrove forests (Frey and Vermeij, 2008; Frey, 2010). A redrawn phylogeny of the study species and their rocky shore ancestry is shown in Fig. 1. *Neripteron violaceum, N. planospira* and *N. balteata* represent three independent transitions, and the consensus is that *Neript. violaceum* is derived from recent freshwater ancestry (Fig. 1; Frey, 2010; Marshall *et al.,* 2015; Wang *et al.,* 2019; Feng *et al.,* 2020, 2021). In all mangrove habitats (shaded trees or shaded or sunned muddy substrata) these snails experience air temperatures of ~25–35°C (Fig. 2; Marshall *et al.,* 2015). On the rocky shores, *N. albicilla* occurs in the low intertidal zone (~0.5–1.0 m Chart Datum, CD), where it is mostly inundated by seawater and thus experiences temperatures ranging between 26 and 31°C (Marshall and McQuaid, 2020). However, the body temperatures of individuals might occasionally rise above 40°C during longer spring tide air exposures. The mid-shore *N. chamaeleon* potentially experiences warmer conditions, though this species appears to behaviourally thermoregulate by seeking cool, shaded refuges under boulders when aerially exposed. Body temperatures of this species probably rise to the upper 40°C range (Fig. 2; Marshall *et al.,* 2015). The high-shore *N. undata* is likely exposed to temperatures approaching 50°C for longer than *N. chamaeleon*. Whereas the rocky shore temperatures vary diurnally between 25°C and 50°C, the mangrove temperatures are relatively stable, daily and seasonally (Fig. 2; Helmuth and Hofmann, 2001; Marshall *et al.,* 2010, 2015; Denny *et al.,* 2011; Brahim *et al.,* 2019).

### Snail collection and laboratory holding

Specimens were collected at low tide from a rocky boulder beach and nearby mangroves in Brunei, between July and December 2017. *Nerita chamaeleon*, *N. undata* and *N. albicilla* were taken from the Empire, along the South China Sea coastline (4.9694°N, 114.8551°E), *N. balteata* and *N. planospira* from the mangroves at Pulau Bedukang, Brunei Bay (4.9795°N, 115.0576°E) and *Neript. violaceum* from mangroves at Sungai Kedayan, a tributary of the Sungai Brunei (4.8981°N, 114.9341°E; Mustapha *et al.,* 2021). More than 15 adult individuals of each species were returned to the laboratory under cool conditions, where they were kept before experiments in an incubator at a constant 27°C on a moist substratum and high humidity for 1–2 days. The experimental snails were thus considered to be field acclimatized.

Additionally, a laboratory temperature-acclimation experiment (common-garden approach) was performed to assess within-individual cTPC trait variation. This experiment used *N. undata* and *Neript. violaceum* snails, which represent the most extreme habitat/evolutionary circumstances. Snails collected between Dec 2022 and Mar 2023 were acclimated for 7–9 days in incubators (Memmert Peltier-cooled, IPP400) set for low temperature acclimation (12 h at 27°C and 12 h at 30°C; n = 12 snails) or high temperature acclimation (6 h rising from 27 to 40°C, 6 h falling from 40 to 27°C, and 12 hours at 27°C; *n* = 12 snails). These thermal regimes represent the natural daily mean temperature variation (Fig. 2). The snails were held in lidded plastic containers under high humidity air and were provided with rocks from their natural environment covered with food substratum (mud/algae). Each day the snails were immersed for 30 mins in estuarine (7–10 psu, *Neript. violaceum*) or open-sea water (30 psu, *N. undata*) to simulate tidal effects and prevent dehydration.

### cTPC and performance attributes

For the primary experiment, cardiac thermal performance was determined for nine individuals of each species, randomly drawn from the collected snail pool. We used six individuals per species for the acclimation experiment. Although more individuals were originally trialled, clear heart patterns were not always achieved due to weak signals and or internal animal movement. These cases were discarded. Shell lengths of the studied snails (mm, mean ± SD) were, *N. chamaeleon* (18.5 ± 1.0), *N. undata* (18.6 ± 1.1), *N. albicilla* (19.0 ± 0.7), *N. balteata* (22.6 ± 1.5), *N. planospira* (19.1 ± 1.1) and *Neript. violaceum* (14.2 ± 0.9). For the temperature-acclimation experiment, shell lengths were, *N. undata* (17.5 ± 0.4) and *Neript. violaceum* (13.5 ± 0.7). Heart rate was measured with optoelectronic infrared sensors adhered to the shells (CNY70, Vishay Semiconductors, Shelton, CT, USA). Signals were amplified, filtered and digitally logged using PowerLab (ADInstruments, Australia) and LabChart 7 (ADInstruments, Australia; Marshall *et al.,* 2011). Sampling rate was set at 40 Hz and amplitude varied between 40 mV and 1000 mV. Heart rates were recorded for snails held in air in plastic bags inside a waterbath that were heated at 0.25°C min^−1^, between 30°C and 65°C. A similar heating rate is naturally experienced on rocky shores (Marshall *et al.,* 2010, 2011). The waterbath temperature was controlled with a programmable Grant TXF200 (Cambridge, United Kingdom). Snail temperatures were simultaneously recorded using calibrated K-type thermocouples adhered to shells and connected to a TC-08 interface and Picolog (Pico Technology, Cambridge, United Kingdom). Analyses were based on raw heartbeat traces or Triangular Barlett smoothed traces (Marshall *et al.,* 2011). The protocol and procedures employed were ethically reviewed and approved by the Faculty of Science Ethics Committee, Universiti Brunei Darussalam.

Physiological temperature attributes were determined for each snail, either directly from the heart rate data or from mathematical descriptions of the profiles (Fig. 3). S*lope gradient*, *Topt* and *HRmax* were assessed from individual cTPCs fitted with a Sharpe-Schoolfield’s model (Schoolfield *et al.,* 1981). The models were fitted to the data using nonlinear least-squares regressions in the R package, *rTPC* (Padfield *et al.,* 2021). Sharpe-Schoolfield’s formulation is process-based, with parameters that can be interpreted biologically. We used the activation energy parameter to represent the *slope gradient* response of each snail. The maximum heart rate estimated for each individual across the entire temperature gradient considered (*HRmax*) and its associated temperature (*Topt*) were extracted from the cTPC model fits. The degree of curvature of the upslope (i.e. *slope curvature*; Fig. 3) was determined based on a second-degree polynomial curve fitted to the upslope section of heart rate vs temperature plots: $HR={c}_0+{c}_1\ast temp+{c}_2\ast {temp}^2$, where ${c}_0$, ${c}_1$ and ${c}_2$ are model coefficients. The second derivative of this equation (i.e. 2 * ${c}_2$) provided a ‘curvature parameter’. The upper lethal thermal trait (*ULT*) was determined from heartbeat flatlining for individuals, which was obtained directly from the recording traces (Fig. 3; Marshall *et al.,* 2010). Notably, while the heartbeat of gastropods sometimes recovers after flatlining following rapid cooling, no studies have shown that snails that have flatlined are capable of emerging from their shells and all typically die within a few days (see also Stenseng *et al.,* 2005; Somero, 2010; Marshall pers. obs.).

### Data and statistical analysis

Statistical analyses were done using R (R Core Team, 2019). To test the predictions that the thermal physiological traits should vary to match habitat conditions (between-habitat responses; Fig. 3) and that this variation should be greater in functional traits, we first determined the values for each of the thermal traits (*ULT*, *Topt*, *HRmax, slope gradient* and *slope curvature*). To examine the effect of habitat on the thermal traits (values averaged by species) while accounting for the species evolutionary relationships, we used a phylogenetic regression analysis and model comparison approach (see Marshall *et al.,* 2015). A phylogenetic tree was built from mitochondrial cytochrome oxidase subunit I sequences retrieved from GenBank (*N. albicilla*, EU732211; *N. bateata*, EU732225; *N. chamaeleon*, EU732227; *N. planospira*, EU732292; *N. violaceum*, MZ831953). Sequences were aligned based on the MUSCLE (MUltiple Sequence Comparison by Log- Expectation) algorithm, and the tree was constructed based on Neighbour Joining method using the software MEGA (Tamura *et al.,* 2021). We used the R package *caper* (Orme *et al.,* 2018) to compute and compare alternative models that made assumptions about the role of phylogeny on the trait responses. We included a ‘star model’ that ignored phylogeny, a Brownian model where phenotypic divergence is proportional to divergence time (phylogenetic signal, λ = 1), and a Pagel model where branch lengths are optimized to maximize the correspondence between phenotypic divergence and divergence time (λ is variable) (Pagel, 1999). To examine within-ecosystem habitat variation, we compared the traits among species for each habitat using generalized linear models (assuming Gaussian error distribution) and Tukey HSD multiple comparison tests. Similarly, for the temperature-acclimation experiment, we examined within-individual trait variation using GLMs followed by Tukey HSD tests. We determined statistical significance based on likelihood-ratio tests, with α = 0.05. For all models, we examined the assumptions of normality and homogeneity of variances visually, based on Normal Q-Q and Residuals vs Fitted Values plots, respectively (Figs S1, S2, S3, S4 andS5).

Although phenotypic variation could reflect greater environmental variation within a habitat, the lack of phenotypic variation implies lack of genetic variation within a population, which influences trait heritability and constrains evolution (Hoffmann and Sgrò, 2011). We calculated the coefficient of variation ($\mathrm{CV}\%$) across species and habitats: $\mathrm{CV}\%=100\left(\mathrm{\sigma} /\mathrm{\mu} \right)$, where σ is the standard deviation and μ the mean. The $\mathrm{CV}\%$ of *Topt* and *ULT* were calculated using temperature data transformed to units of Kelvin.

## Results

**Figure 4:**
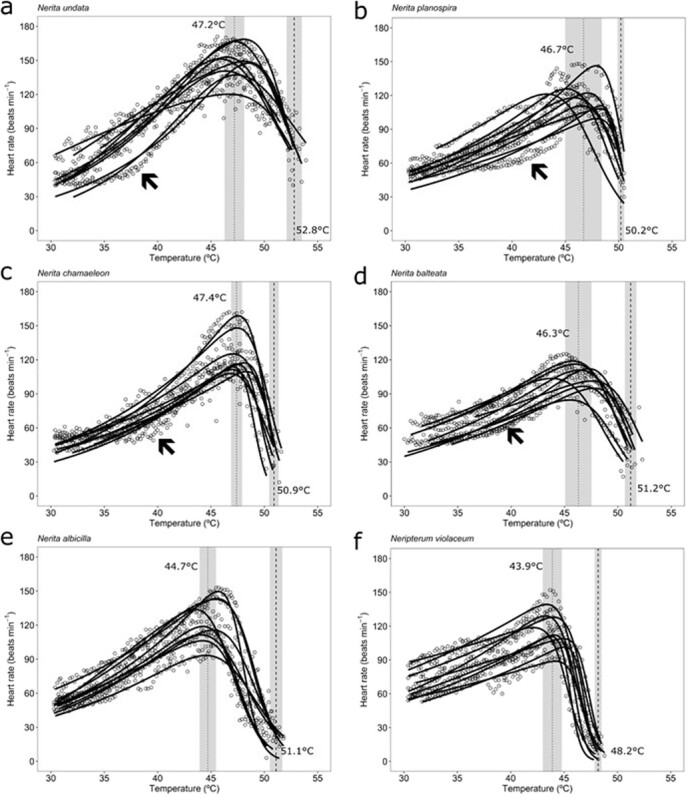
Variability on cTPC described using the Sharpe-Schoolfield’s model for rocky shore snails (a = *N. undata*, c = *N. chamaeleon* and e = *N. albicilla*) and mangrove snails (b = *N. planospira*, d = *N. balteata* and f = *Neript. violaceum*). Dotted and dashed vertical lines represent mean (± SD, shaded area) values of *Topt* and mean *ULT*, respectively. *n* = 9 snails for each species. Arrows indicate thresholds for temperature-insensitive heart rate responses by some individuals.

**Figure 5:**
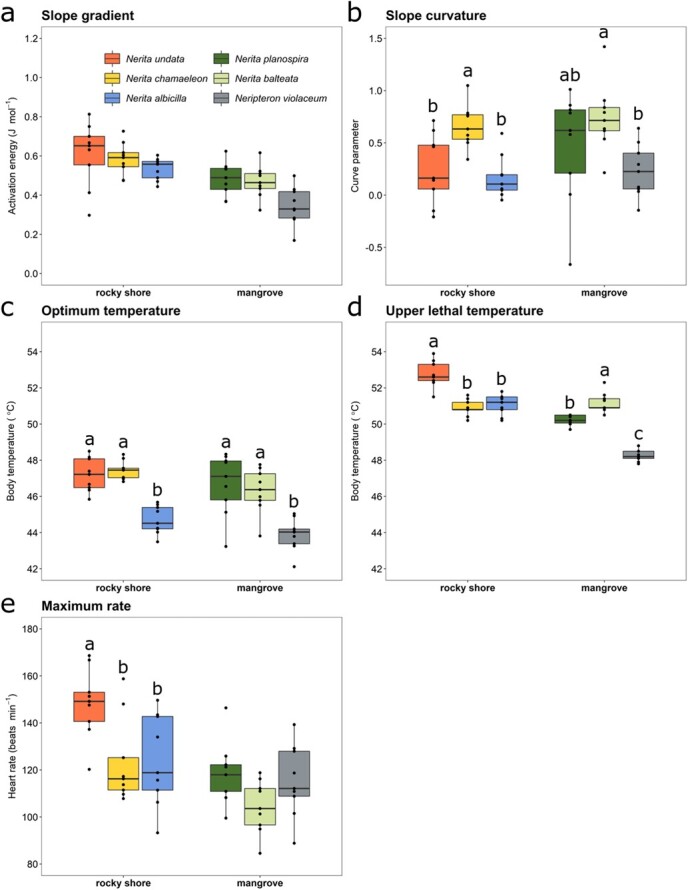
Physiological thermal traits estimated from TPCs (heart rate versus temperature) for three rocky shore and three mangrove neritid snail species. (a) and (b) Slope parameter of the relationship between heart rate (log_2_-transformed) and temperature for the up-slope section of the curves (i.e. temperatures < *Topt*). (c) Optimal temperatures (*Topt*). (d) Upper lethal temperature (*ULT*). (e) Maximum heart rate (*HRmax*). The *slope gradient* and *Topt* represent sub-lethal traits, while *ULT* is the lethal physiological limit. Different letters above the boxes indicate habitat-specific differences among species.

The cTPCs were typically unimodal, and generally well captured by Sharpe-Schoolfield’s models. However, individuals of some species (*N chamaeleon*, *N. undata*, *N. planospira* and *N. balteata*) showed greater flattening of this curve over the lower temperature range (< 40°C) compared with others (*N. albicilla* and *Neript. violaceum*) (Fig. 4). Mean maximum heart rate (*HRmax*) varied across the species between 108 and 151 bpm (Fig. 5).

Regarding the between-habitat comparisons, we found that *slope gradient* was higher for rocky shore than mangrove species (*P* = 0.04, Fig. 5, Tables 1, 2). All other thermal traits were independent of habitat (Tables 1, 2). The model comparison approach also revealed that the phylogenetic signal was important for describing the *slope gradient* (*wAIC_c_* = 0.56), the *slope curvature* (*wAIC_c_* = 0.52), and *Topt* (*wAIC_c_* = 0.52) (Table 1). In contrast, the top-ranked models for *HRmax* and *ULT* did not include a phylogenetic signal (λ = 0). The Pagel models, where the phylogenetic signal (λ) is estimated, were poorly supported (*wAIC_c_* = 0, Table 1).

**Table 1:** Comparison between linear regression models describing the effect of habitat on three cTPC traits

Model	Statistics	Phylogenetic signal λ	*AIC_c_*	*wAIC_c_*
*Slope gradient ~ habitat*	*t_6_* = 2.84, *P* = 0.04	0	9.10	0.44
*Slope gradient ~ habitat*	*t_6_* = 2.84, *P* = 0.00	0.95	37.43	0.00
*Slope gradient ~ habitat*	*t_6_* = 2.84, *P* = 0.04	1	8.62	0.56*
*Slope curvature ~ habitat*	*t_6_* = −0.57, *P* = 0.60	0	20.73	0.48
*Slope curvature ~ habitat*	*t_6_* = −0.99, *P* = 0.38	< 0.001	50.54	0.00
*Slope curvature ~ habitat*	*t_6_* = −0.90, *P* = 0.42	1	20.56	0.52*
*HRmax ~ habitat*	*t_6_* = 2.07, *P* = 0.11	0	50.98	0.56*
*HRmax ~ habitat*	*t_6_* = 0.70, *P* = 0.52	< 0.001	78.27	0.00
*HRmax ~ habitat*	*t_6_* = 2.18, *P* = 0.09	1	51.48	0.44
*Topt ~ habitat*	*t_6_* = 0.65, *P* = 0.56	0	34.91	0.48
*Topt ~ habitat*	*t_6_* = 0.16, *P* = 0.88	< 0.001	64.19	0.00
*Topt ~ habitat*	*t_6_* = 0.54, *P* = 0.62	1	34.72	0.52*
*ULT ~ habitat*	*t_6_* = 0.96, *P* = 0.39	0	42.00	0.53*
*ULT ~ habitat*	*t_6_* = 1.48, *P* = 0.21	< 0.001	71.93	0.00
*ULT ~ habitat*	*t_6_* = 0.59, *P* = 0.58	1	42.23	0.47

Habitat-specific models (between-species responses) revealed significant differences (linear models; *P* < 0.05 for all tests except *slope gradient* for rocky shore and *HR_max_* for mangrove), with relatively similar patterns across traits (Fig. 5). For the rocky shore, attribute values were typically higher for *N. undata* and lowest for *N. albicilla*. *N. chamaeleon* was generally intermediate, although it showed the highest *slope curvature*. For mangrove species, values were typically higher for *N. planospira* and *N. balteata*, and lowest for *Neript. violaceum* (Fig. 5).

**Figure 6:**
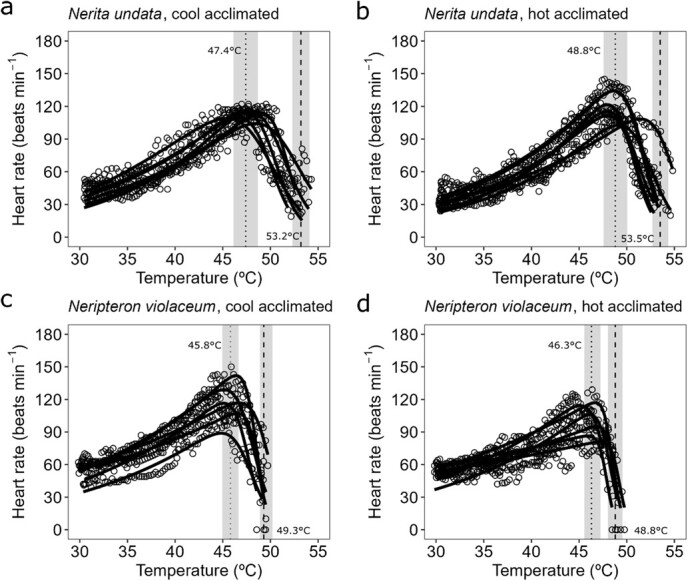
Acclimation experiment. Variability on cTPC described using the Sharpe-Schoolfield’s model for a rocky shore snail (*Nerita undata*) and a mangrove snail (*Neripteron violaceum*) acclimated to cool and hot temperature treatments. Dotted and dashed vertical lines represent mean (±SD, shaded area) values of *T*_*op*t_ and mean *ULT*, respectively. *n* = 6 snails for each species.

**Figure 7:**
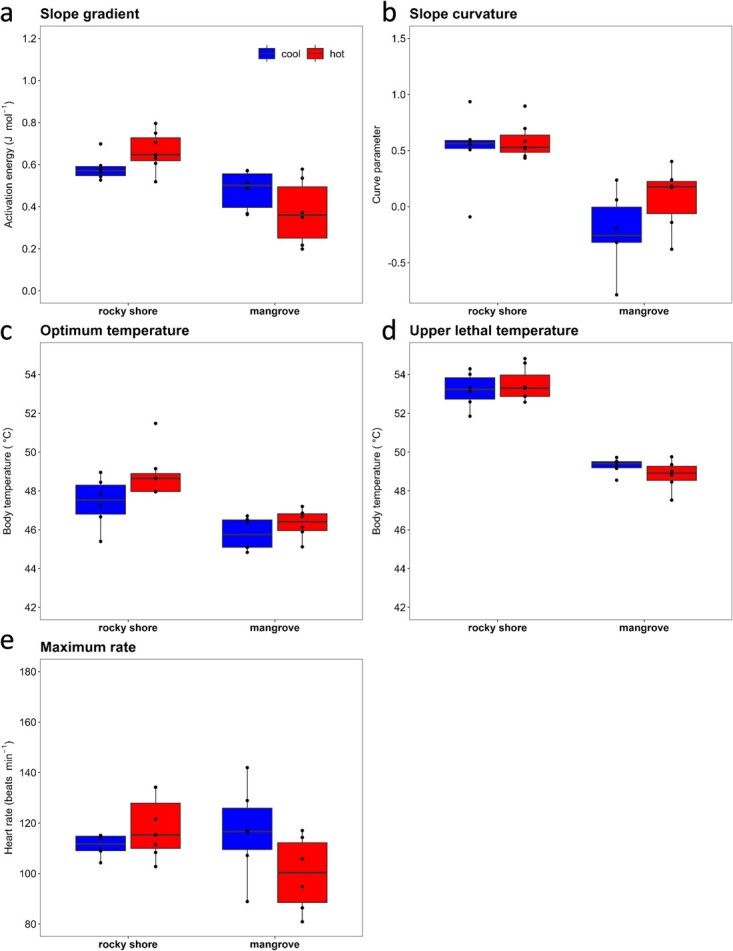
Acclimation experiment. Physiological thermal traits estimated from TPCs (heart rate versus temperature) for a rocky shore (*N. undulata*) and a mangrove neritid (*Neript. violaceum*) snail species acclimated to hot and cool temperature treatment. (a) *Slope gradient* and (b) *slope curvature* parameters of the relationship between heart rate and temperature for the up-slope section of the curves (i.e. temperatures < *Top*t). (c) Optimal temperatures (*Top*t). (d) Upper lethal temperature (*ULT*). (e) Maximum heart rate (*HRmax*). The *slope gradient*, *slope curvature* and *Top*t represent sublethal traits, while *ULT* is the lethal physiological limit. Different letters above the boxes indicate habitat-specific differences between species.

The acclimation experiment revealed a minimal influence of acclimation temperature on the traits for both the rocky shore and the mangrove species considered, *N. undata* and *Neript. violaceum* (Figs 6 and7). The only significant effect identified suggests a slightly higher *ULT* for the mangrove species acclimated to a cool than warm temperature (mean ± SD = 51.3 ± 1.0 and 50.1 ± 0.52°C, respectively; LRT, χ^2^ = 6.36, df = 1, *P* = 0.012; Fig. 7d). The temperature acclimation experiment also allowed comparing the traits between cool- and warm-acclimated, and field-fresh (primary experiment) individuals of *N. undata* and *Neript. violaceum*. For *N. undata*, we found that *ULT* of field-fresh individuals was slightly higher than that of cool- (LRT, χ^2^ = 11.31, df = 2, *P* = 0.004; Tukey HSD, *P* < 0.001) and warm-acclimated (Tukey HSD, *P* < 0.001) animals. The *HRmax* of field-fresh *N. undata* was extraordinarily higher than that of cold- (LRT, χ^2^ = 41.77, df = 2, *P* < 0.001; Tukey HSD, *P* < 0.001) and warm-acclimated ones (Tukey HSD, *P* < 0.001; see also Figs 4 and6). The *slope gradient*, *slope curvature* and *Topt* were unaffected by acclimation (Tukey HSD, *P* > 0.05 in every case). For *Neript. violaceum* we found that acclimation treatments modified the trait values, relative to those of field-fresh individuals. The *slope gradient* was higher for field-fresh than hot-acclimated animals (LRT, χ^2^ = 9.50, df = 2, *P* = 0.008; Tukey HSD, *P* = 0.007). The *slope curvature* (LRT, χ^2^ = 8.70, df = 2, *P* = 0.013; Tukey HSD, *P* = 0.009) and *Topt* (LRT, χ^2^ = 10.26, df = 2, *P* = 0.006; Tukey HSD, *P* = 0.005) were higher for field-fresh than hot-acclimated animals. In turn, the *ULT* and *HRmax* of field fresh *Neript. violaceum* were higher than for both cool- (*ULT*; LRT, χ^2^ = 90.82, df = 2, *P* < 0.001; Tukey HSD, *P* < 0.001; *HRmax*; LRT, χ^2^ = 36.22, df = 2, *P* < 0.001; Tukey HSD, *P* < 0.001) and hot-acclimated individuals (*ULT*; Tukey HSD, *P* < 0.001; *HRmax*; Tukey HSD, *P* < 0.001).

**Table 2:** Summary of relative responses of cTPC traits

Trait type	Trait	Ecosystem comparison	Species comparison	
Functional	*Slope gradient*	RS > M	Nu = Nc = Na	Np = Nb = Nv
	*Slope curvature*	RS = M	Nu < Nc > Na	Nb > Nv = Np; Nb = Np
	*HRmax*	RS = M	Nu > Nc = Na	Np = Nb = Nv
	*Topt*	RS = M	Nu = Nc > Na	Np = Nb > Nv
Lethal limit	*ULT*	RS = M	Nu > Nc = Na	Nb > Np > Nv

**Figure 8:**
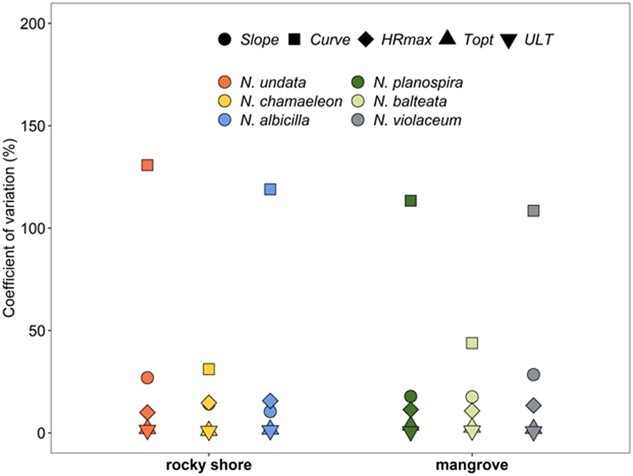
Coefficient of variation ($CV\%$) of the sub-lethal (i.e. *Topt*), lethal (i.e. *ULT*) and performance (i.e. *slope gradient*, *slope curvature*, and *HRmax*) thermal traits of three rocky shore and three mangrove neritid gastropod species.

Regarding between- and within-trait variability, the least variable thermal attributes across habitats were *ULT* (CV; mean ± SD = 0.955 ± 0.292) and *Topt* (CV; mean ± SD = 2.19 ± 0.88), followed by the *HRmax* (CV; mean ± SD = 12.6 ± 2.28) and the *slope gradient* (CV; mean ± SD = 19.2 ± 7.11) (Fig. 8). The *slope curvature* was the most variable thermal trait (CV; mean ± SD = 91.1 ± 42.4). This hierarchy was consistent for both habitats (Figs 5 and8; Table 2). The observation that the lethal trait *ULT* was the least variable among individuals suggests constrained selection of this trait.

## Discussion

Neritid snails that evolutionarily transitioned to cool mangrove environments and currently experience vastly cooler habitat temperatures were found to differ little in thermal physiology from their warmer rocky shore counterparts. Our analysis accounting for phylogenetic relatedness, showed limited difference in most cTPC traits between the intertidal ecosystems (Table 1). *Slope gradient* was an outlier, such that the mangrove species produced flatter slopes than field-fresh rocky shore species during acute experimental warming. Slope flattening of the mangrove species could be seen as a directional change for life in a cooler environment (see Fig. 2). This can be explained as either adaptive selection to better match the thermal physiology with the environmental temperature (Angilletta, 2009), or to reduce the cost of maintaining redundant thermal tolerance traits (Feder and Hofmann, 1999). Alternatively, this may represent a non-adaptive random loss of a plesiomorphy that evolved for life on the thermally more-variable and warmer rocky shores (evolutionary drift). Notably, however, the thermal acclimation experiment showed unique flattening of the otherwise steeper slopes in rocky shore snails. Such thermal plasticity appears to allow individuals to modulate their physiological machinery in favour of homeostasis (energetic cost saving) when resting for long periods under high temperature exposure on rocky shores (Newell, 1969; Newell and Branch, 1980; Marshall and McQuaid, 2011; Marshall *et al.,* 2013).

For each ecosystem separately, the maximum heart rate (*HRmax*) and optimum temperature for performance (*Topt*) differed significantly among the species in expected ways relative to habitat temperature variation (Fig. 5). The lowest *Topt* values were recorded in *N. albicilla*, which occupies the coolest, lower rocky shore zone, and in *Neript. violaceum*, which is ancestrally associated with cooler freshwater ecosystems. The unique elevation of maximal heart rate in *N. undata* coincides with its hot, upper rocky shore occupation (Fig. 5; Monaco *et al.,* 2017). *Slope curvature* was uninformative across the ecosystems, habitats and species, with no clear pattern emerging. Cardiac rate-temperature relationships of intertidal gastropods are known to deviate from predictions for ectothermic animals in general, due to induced temperature-insensitive metabolic depression when resting (see Fig. 3; Newell, 1969; Newell and Branch, 1980; Marshall *et al.,* 2013; Marshall and McQuaid, 2011; Verberk *et al.,* 2016). This allows for the conservation of energy resources when feeding becomes limited, and is especially common in higher-shore gastropods, which experience severe feeding constraints (due to prolonged inactivity) under extreme heating (Marshall and McQuaid, 2011; Marshall *et al.,* 2013). Initial plateauing of the cTPC was observed in *N. undata* (one individual), *N. chamaeleon* (three individuals), *N. balteata* (three individuals) and *N. planospira* (four individuals; Figs 3 and5), suggesting the retention of temperature-induced metabolic depression in the mangrove species, despite this having no adaptive benefit to them. Such individual variability in cardiac depression is commonly observed in marine gastropod study cohorts apparently comprising individuals that differ in their instantaneous energy demand (see Marshall and McQuaid, 2011, 2020).

Heat tolerance (*ULT*) was found to relate to habitat conditions and evolutionary background. While *N. undata* (the rocky shore species that lives in one of the hottest environments globally; Somero *et al.,* 2017) exhibited the greatest heat tolerance, *Neript. violaceum* (the mangrove species that underwent a uniquely cool freshwater evolutionary incursion) displayed the lowest heat tolerance. These observations imply thermal specialization in *N. undata* snails and loss of heat resistance in *Neript. violaceum*. Although directional selection is likely muted in *Neript. violaceum* as no obvious benefit is derived from reducing heat tolerance in the cooler mangrove environment, notably, the maintenance of this resistance trait is not without a cost (see above; Feder and Hofmann, 1999). The most closely-related neritid species pair, marking the most recent ecosystem transition, the rocky shore *N. chamaeleon* and the mangrove *N. balteata*, present a useful case for comparison. These species have identical heat tolerance capabilities, despite experiencing very different contemporary thermal regimes and ecological divergence over ~40 mya, when global temperatures were ~3°C warmer (Figs 1 and5; Table 2; Feng *et al.,* 2021). The phenotypic variation among individuals of a population indicates the potential for selection (Hoffmann and Sgrò, 2011). The lowest value of the coefficient of variation was recorded for the *ULT*, suggesting resistance of this trait to change, in compliance with the general pattern found in animal ectotherms (Fig. 8; Araújo *et al.,* 2013).

Our key finding (in the primary experiments) of limited thermal physiological difference between the ecosystems was observed despite not accounting for the potentially confounding effect of acclimatization to the different thermal regimes in the rocky shore and mangrove ecosystems. A separate common-garden experiment (acclimating the species from the most extreme habitats in each ecosystem, *N. undata* and *Neript. violaceum*, to rocky shore or mangrove mean temperatures) revealed that cardiac trait values were largely unaffected by laboratory acclimation. The most striking outcome of these experiments was, however, the difference in *HRmax* between acclimated and field-fresh *N. undata* snails. This effector of *slope gradient* was markedly depressed in both cool- (0.75-fold decrease) and warm-acclimated *N. undata* (0.80-fold decrease) individuals (Figs 5 and7). This could be due to the less than optimal laboratory holding conditions, or snails not being exposed to the higher range of temperatures (> 40°C) commonly experienced on rocky shores. Relatively minor differences were observed between field-fresh and acclimated responses in the mangrove species, *Neript. violaceum*, suggesting loss of acclimatory capacity in the thermally more stable environment. Overall, the acclimation experiment highlighted the role of thermal plasticity in negating the earlier observation (primary experiment) of *slope gradient* differences (see above)*.* Despite the preponderance of ectothermic animals to have relatively fixed heat tolerances (*ULT*; Araújo *et al.,* 2013), the *ULT*s of tropical intertidal gastropods are often flexible, due to heat-hardening under stressfully high temperatures (usually above mid-40°C; Brahim *et al.,* 2019); However, we found no difference in the *ULT* of the differently-acclimated snails, for both species, which again probably relates to the relatively benign thermal regimes used in the common-garden experiment (< 40°C).

What do the observed cardiac thermal performance patterns mean in terms of functional adaptation? It is commonly regarded that gastropod cardiac performance mirrors cellular oxygen demand and therefore cTPCs can be interpreted with reference to organismal functionality (Marshall *et al.,* 2011). Such interpretation is, however, complicated here by the different thermal regimes of the ecosystems (Fig. 2), and by the mismatch generally in intertidal gastropods of thermal performances for locomotor activity and physiology (Monaco *et al.,* 2017). Because locomotor activity of intertidal gastropods ceases at temperatures well below those tolerated when resting (*CTmax* is often by as much as 10°C below the *ULT*), cardiac performance above the *CTmax* (typically between 40 and 45°C in tropical species) must be unrelated to energy aquisition (see Monaco *et al.,* 2017; Ørsted *et al*. 2022). This means that the cardiac activity in the higher thermal range considered here (>  40°C) is primarily associated with energetic support of a heat shock response in the rocky- shore neritid snails, but must be redundant in the mangrove snails that never naturally experience these higher temperatures. Thus, whereas the *HRmax* and *Topt* values in the rocky- shore species likely refer to heat tolerance adaptation, in the mangrove species they refer to a non-adaptive thermodynamic effect (Angilletta, 2009). Because the locomotor activity of intertidal gastropods is not only inherently limited by morphology, but is also confined to the narrow and cool temperatures of the range tolerated (Monaco *et al.,* 2017), the role of thermal physiology in enhancing fitness through energy acquisition is likely under weak evolutionary selection. Rather, thermal adaptation relating to the energetics of these gastropods likely mainly concerns energy utilization during inactivity, particularly the servicing of the contrasting demands of conserving energy resources and supporting heat tolerance (i.e., a heat shock response). Notably again, the latter is irrelevant to the mangrove species.

What are the implications of conserved or redundant physiology with respect to species resilience to climate change? Functional thermal redundancy in the neritid snails is further suggested by the magnitude of the temperature difference between the habitats and the ecosystems studied. Habitat thermal regimes can be simply described in terms of mean and extreme temperatures, and most studies suggest that functional physiologies adaptively respond to mean temperature variation (Bozinovic *et al.,* 2011; Barker *et al.,* 2021). The difference in mean temperature between the rocky shore and mangrove ecosystems is only ~4°C, whereas the maximum (extreme) temperatures driving heat tolerance selection vary by as much as ~20°C (Fig. 2). These differences suggest that more frequent and severe heatwaves predicted during warmer climates are more likely to impact neritid snails (and other intertidal snails) through their effect on heat tolerance rather than energetics. Physiological trait conservation and hence mismatching of *ULT* with environmental temperature, should lead to significant thermal buffering of climate warming by the mangrove species. More specifically, these species should have extended warming tolerance values (*WT* = *ULT* minus daily Te_max_; Deutsch *et al.,* 2008; but also see Clusella-Trullas *et al.,* 2021). These circumstances suggest that the forests could act as thermal refugia for other mangrove gastropods (see Marshall *et al.,* 2015), assuming that the forests themselves are resilient to climate change. The conservation of mangroves forests is crucial for the local existence of such gastropod communities. Additionally, such a mismatch between environmental temperature and heat tolerance presents a caveat in the general use of climate envelope (or species distribution) modelling, which is based on the assumption of adaptation to contemporary thermal niches (Huey *et al.,* 2012).

## Conclusions

The thermal physiologies of the neritid snail species were similar and unrelated to habitat temperature variation, following a lineage transition from rocky shores to mangroves. A single cardiac thermal performance parameter (*slope gradient*) differed between the ecosystems, but this difference was negated when taking into account thermal plasticity. Although we predicted greater ability of functional cTPC traits (capacity) compared with the trait limiting all performance (tolerance), interpretations need to consider the thermal ranges for locomotor performance, as well as the temperatures naturally experienced. Doing so, revealed that the cardiac functional trait responses of these snails, and probably most intertidal gastropods, primarily involve supporting heat tolerance, rather than energy acquisition. These nuanced observations can be insightful, and can guide new approaches to assessing organismal responses to climate change. Interestingly, they contradict a mainstream metaanalytical conclusion that heat tolerance is unrelated to climate ancestry (Bennett *et al.,* 2021). The mismatching of conserved physiologies with novel cooler environments that buffer otherwise lethal climate warming exposure, carries implications for the use of niche modelling to predict species responses to climate warming (Kearney and Porter, 2009). It is important to know how broadly the circumstances outlined in this study extend across taxa and ecosystems, in order for the possible future inclusion of evolutionary information in assessments of global warming responses. 

## Authorship

All authors wrote the paper, made a substantial direct and intellectual contribution to the work and approved it for publication. Reviewers’ comments led to an improved version of the manuscript.

## Competing interests

The authors declare no competing interests.

## Funding

This work was supported by Universiti Brunei Darussalam, through a grant to DJM (UBD/RSCH/1.4/FICBF(b)/2021/033).

## Data availability

Data will be provided on request to the first author (D.J.M.).

## Supplementary Material

Web_Material_coad056
