# Supplementary material for: Conservation of thermal physiology in tropical intertidal snails following an evolutionary transition to a cooler ecosystem: climate change implications
Source: Conserv Physiol. 2023 Aug 1;11(1):coad056. doi: 10.1093/conphys/coad056 (PMC10393397; doi:10.1093/conphys/coad056)
Supplement: Web_Material_coad056 [file web_material_coad056.pdf]

Supplementary materials

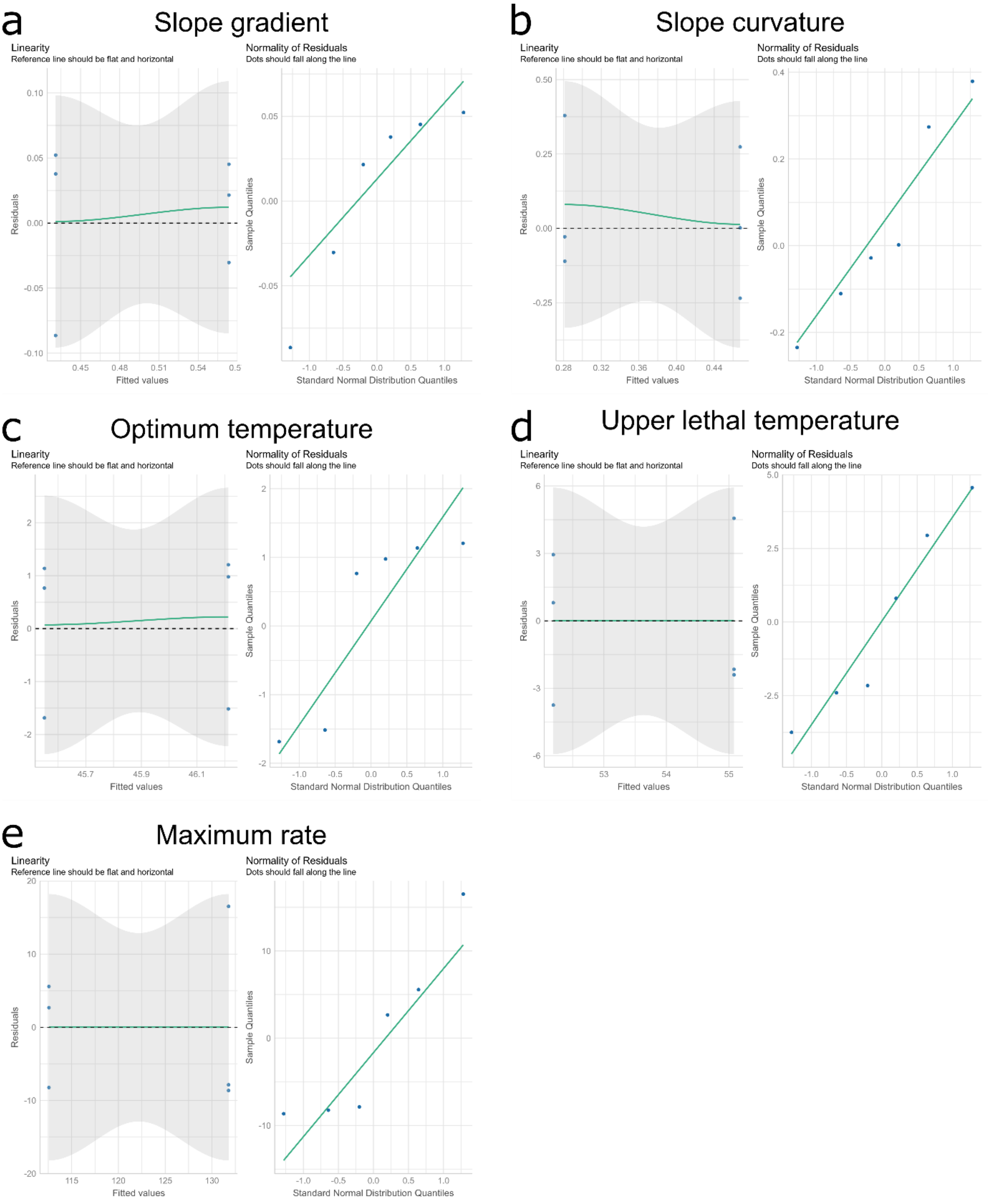

Fig. S1 Model-diagnostic plots to evaluate normality (QQ-plots) and homogeneity of variance (fitted data versus residuals) in the linear mixed effects models used to test the effect of habitat on thermal performance traits. Panels (a), (b), (c), (d), and (e) are for the *slope gradient*, *slope curvature*, T_{opt}, *ULT*, and HR_{max} models, respectively. Overall, the absence of obvious patterns indicates that models were adequate.

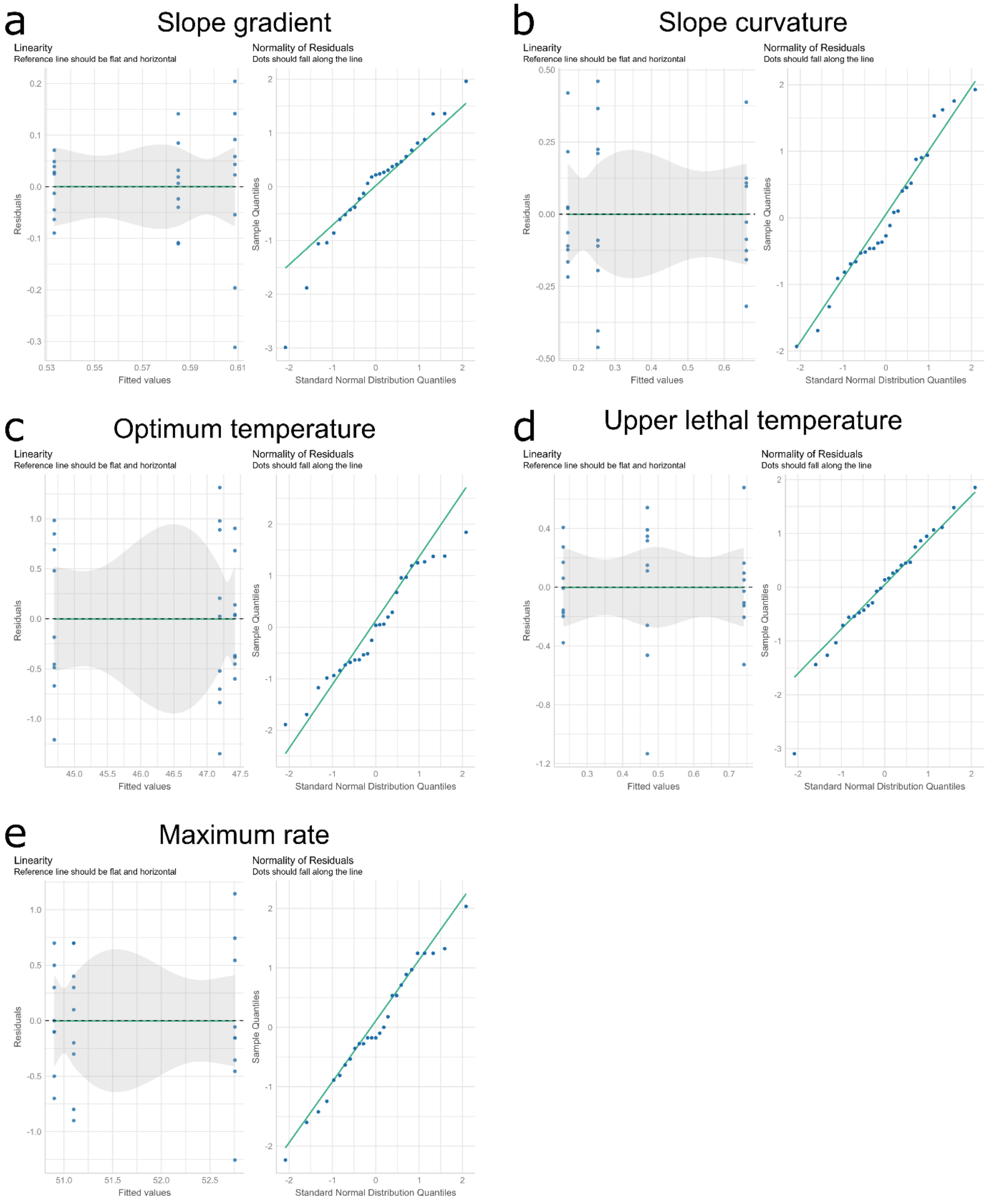

Fig. S2 Model-diagnostic plots to evaluate normality (QQ-plots) and homogeneity of variance (fitted data versus residuals) in the generalized linear models used to test the effect of rocky-shore species on thermal performance traits. Panels (a), (b), (c), (d), and (e) are for the *slope gradient*, *slope curvature*, T_{opt}, HR_{max}, and *ULT* models, respectively. Overall, the absence of obvious patterns indicates that models were adequate.

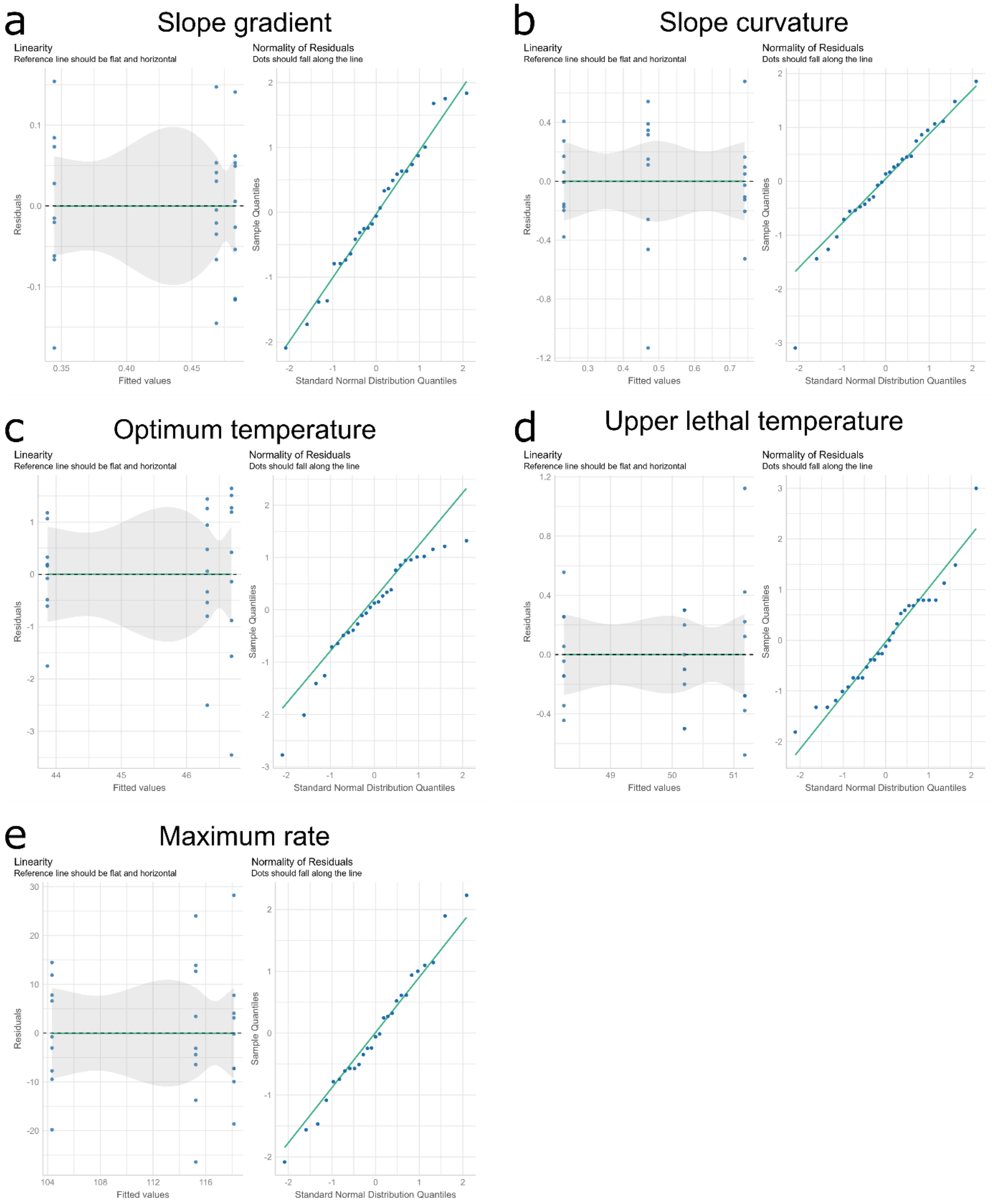

Fig. S3 Model-diagnostic plots to evaluate normality (QQ-plots) and homogeneity of variance (fitted data versus residuals) in the generalized linear models used to test the effect of mangrove species on thermal performance traits. Panels (a), (b), (c), (d), and (e) are for the *slope gradient*, *slope curvature*, T_{opt}, HR_{max}, and *ULT* models, respectively. Overall, the absence of obvious patterns indicates that models were adequate.

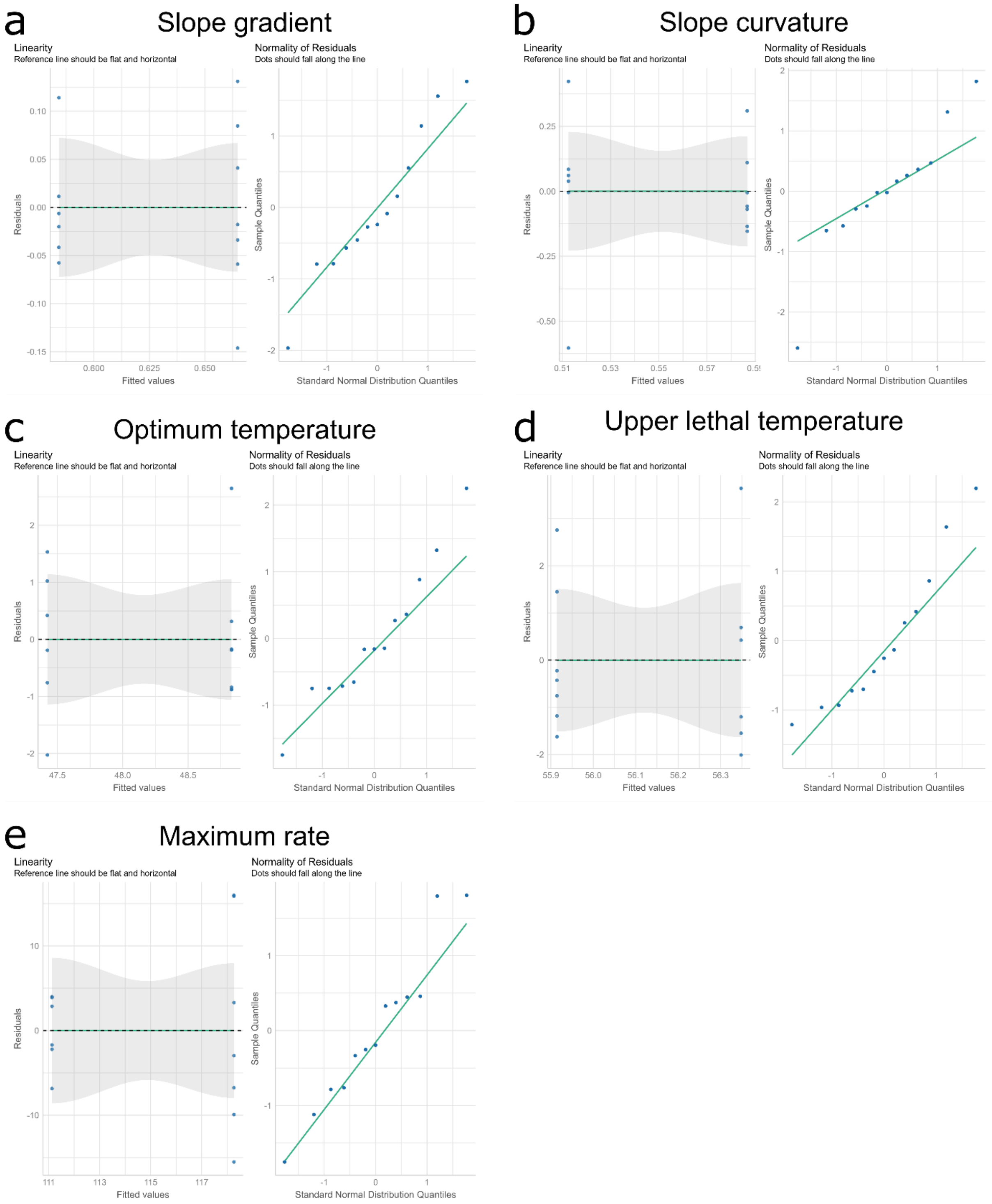

Fig. S4 Model-diagnostic plots to evaluate normality (QQ-plots) and homogeneity of variance (fitted data versus residuals) in the generalized linear models used to test the effect of acclimation temperature on thermal performance traits of the rocky-shore species (*Nerita undata*). Panels (a), (b), (c), (d), and (e) are for the *slope gradient*, *slope curvature*, T_{opt}, *ULT* and HR_{max} models, respectively. Overall, the absence of obvious patterns indicates that models were adequate.

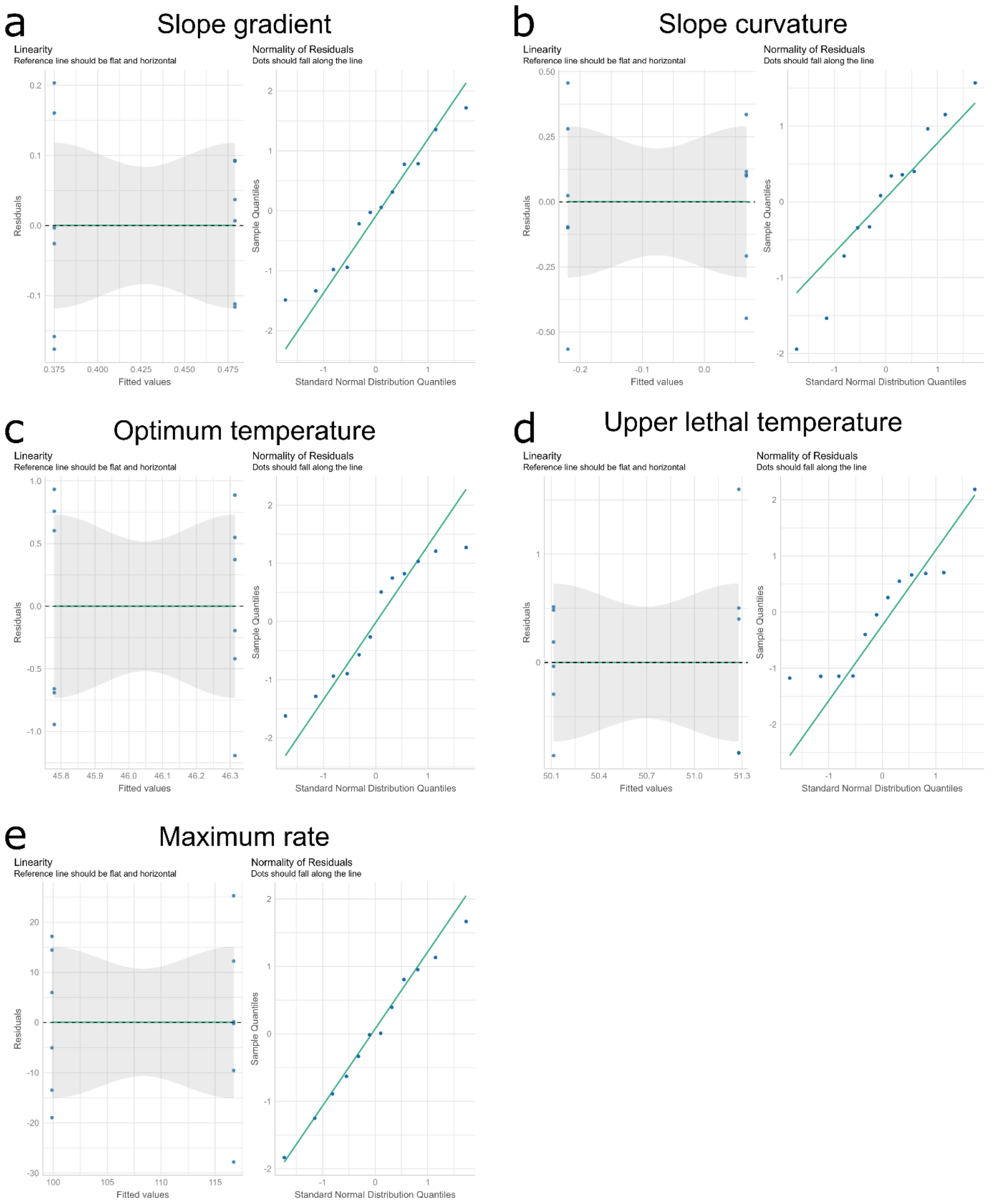

Fig. S5 Model-diagnostic plots to evaluate normality (QQ-plots) and homogeneity of variance (fitted data versus residuals) in the generalized linear models used to test the effect of acclimation temperature on thermal performance traits of the mangrove species (*Neripteron violaceum*). Panels (a), (b), (c), (d), and (e) are for the *slope gradient*, *slope curvature*, T_{opt}, *ULT* and HR_{max} models, respectively. Overall, the absence of obvious patterns indicates that models were adequate.
